# The Association between Migraine and Types of Sleep Disorder

**DOI:** 10.3390/ijerph15122648

**Published:** 2018-11-26

**Authors:** Seung Ju Kim, Kyu-Tae Han, Suk-Yong Jang, Ki-Bong Yoo, Sun Jung Kim

**Affiliations:** 1 Institute of Health Services Research, Yonsei University, Seoul 03722, Korea; seungju.phd@gmail.com (S.J.K.); kthan.phd@gmail.com (K.-T.H.); 2 Department of Nursing, College of Nursing, Eulji University, Seongnam 13135, Korea; 3 Department of Policy Research Affairs, National Health Insurance Service Ilsan Hospital, Ilsan 10444, Korea; 4 Department of Preventive Medicine, School of Medicine, Eulji University, Daejeon 34824, Korea; sukyong@eulji.ac.kr; 5 Department of Health Administration, College of Health Sciences, Yonsei University, Wonju 26493, Korea; kb53545@gmail.com; 6 Department of Health Administration, Soonchunhyang University, Chungnam 31538, Korea

**Keywords:** sleep disorder, migraine, sleep disturbance, headache, accessibility

## Abstract

*Background*: Migraines gradually increase year by year, as does its burden. Management and prevention are needed to reduce such burdens. Previous studies have suggested that daily health behaviors can cause migraines. Sleep is a substantial part of daily life, and in South Korea, the average sleep duration is shorter than in other countries. Thus, this study focused on the increase of both diseases, and analyzed sleep disorders as a risk factor for migraines. *Methods*: The data used in this study was that of the national health insurance service (NHIS) national sample cohort. We used a matched cohort study design that matched non-patients based on patients with sleep disorders, and included 133,262 patients during 2012–2015. We carried out a survival analysis using a Cox proportional hazard model with time-dependent covariates to identify the association between migraines and sleep disorders. *Results*: Approximately 11.72% of patients were diagnosed with migraines. Sleep disorders were positively correlated with the diagnosis of migraine (Hazard Ratio, 1.591; *p* < 0.0001). By the types of sleep disorder, patients who were diagnosed as having insomnia, rather than other types of sleep disorder, had the greatest associations with migraine. The associations were greater for males, people with lower income, the elderly population, and patients with mild comorbid conditions. *Conclusion*: This study provides evidence that migraine is associated with sleep disorders, especially insomnia. Based on these findings, healthcare professionals and policy makers have to reconsider the present level of insurance coverage for sleep medicine, recognize the risk of sleep-related diseases and educate patients about the need for appropriate care.

## 1. Introduction

A migraine is defined as a recurrent, moderate to severe headache on one side of the head, during which patients cannot participate in regular daily activities for least 4 h [1,2]. It is categorized as episodic (<15 headache days per month) and chronic (≥15 headache days per month), based on the frequency of the headache, and can be classified as having or not having aura [3,4]. In general, patients with migraines would experience the following symptoms: nausea, vomiting, and sensitivity to light, smell, or sound [5]. About 23% of households contained at least one member who suffered from migraines, most of which could be controlled by simple medication, but frequent use of medication may result in its overuse, leading to additional severe pain [6,7,8]. In addition, in daily life, migraines are a risk factor for psychiatric problems, such as depression, or for a reduced quality of life [9,10].

The report of the National Health Insurance Service (NHIS) shows that the prevalence of migraines has gradually increased annually (2010: 488,733; 2016: 535,305), and its financial burden has also increased (23 million dollars to 37 million dollars) [11]. It is expected that various health problems will increase with these trends, and that proper management and methods to prevent migraines are needed. Although several factors that can cause migraines, including genetic or remediable factors, such as obesity and caffeine overuse, were analyzed in previous studies, a clear cause has not yet been identified [12,13,14]. Excluding such factors, daily health behavior can have a role in causing migraines, according to previous studies [15]. In this study, we focused on the association with sleep disorders as a risk factor for migraines.

Sleep is a substantial part of daily life, and it is slowly gaining the spotlight as a common risk factor for physical or psychiatric health problems [16,17]. Therefore, many health professionals have paid attention to sleep, and have analyzed sleep dynamics, including cardiovascular disease and its association with depression [18,19]. Those studies have helped people to understand the importance of optimal sleep duration and quality. Nevertheless, given the social atmosphere in South Korea, people are unable to manage their sleep health by themselves, because social and economic competition is greater than it was in the past, even though such competitiveness has rapidly improved. According to Organization for Economic Cooperation and Development (OECD) countries, the average sleep time in South Korean was lower than the OECD average (South Korea: 462 min per day; OECD average: 502 min per day) [20]. A decrease in sleep time reflects a decrease in actual sleep time due to overwork, or difficulty sleeping or maintaining sleep [21]. These sleep disturbances reflect poor sleep quality, and can cause psychiatric disorders, such as insomnia [22,23]. Sleep disorders include a variety of disorders, such as sleep-related breathing disorders, as well as insomnia [24]. The NHIS reported that the number of patients with sleep disorder is rapidly increasing each year (2010: 287,835; 2016: 494,915) [25]. There have been studies on sleep disorders that affect other diseases, but this problem is especially regarded as a trigger factor for migraine [26]. Sleep deprivation or sleep overload can lead to migraine, and low melatonin concentrations has been reported in both patients with migraine and those with insomnia [27]. Migraine patients experienced pain due to lack of sleeping duration and sleep quality, and the pain improved with sleep [28]. In case control studies, patients with migraine tend to complain of sleep deprivation, and excessive weekly sleepiness has been more frequent than in the general population [29]. Study have shown that patients with severe headaches, including migraine with or without aura, have difficulty falling asleep [30]. Although the direction of the relationship between migraine and sleep is unknown, research has shown that interventions in sleep habits can improve headaches in children [31]. So far, there are many epidemiological findings related to sleep disorders. A common concept is that sleep disorders lead to other physical or psychiatric disorders because of insufficient rest during bedtime [17].

Studies have shown that poor quality or poor duration of sleep can result in headaches, including migraines, but in most cases, the study was conducted on a Western population and the sample size was small [32,33]. Asian studies on the relationship between sleep and migraine are lacking, and evidence is needed, based on representative samples. Thus, the aim of this study was to suggest evidence on the association between organic sleep disorder and migraines, in the hopes that more developed strategies for managing new disease paradigms, including increases of sleep disorder or migraines, will be initiated.

## 2. Methods

### 2.1. Study Population

The data used in this study was from the NHIS National Sample Cohort 2002–2015, which was released by the NHIS in 2017. These data included 1,000,000 representative individuals, who were randomly selected from the South Korean population in 2006. The data include all medical claims filed from January 2002 to December 2015. We first excluded all patients who were deceased, and then identified patients who were diagnosed with migraines (International Classification of Diseases [ICD]-10: G43) or sleep disorders (ICD-10: G47). We assumed that these patients were newly diagnosed with migraines or sleep disorders, excluding patients before 2004. We excluded patients who were diagnosed with migraines before sleep disorders. Finally, we identified 66,631 patients with sleep disorders over 2004–2015. To design the matched cohort study using the sleep-disorder patients as the case group, we established the control group by propensity score matching by adjusting for similar sex, age (5-year intervals), and observation periods. We used a 1:1 matching method to select the control group (66,631 patients who were not diagnosed with sleep disorders). Finally, the data used in this study contain the records of 133,262 patients over 2004–2015.

### 2.2. Variables

The dependent variable used in this study was whether patients were newly diagnosed with migraines during the study period. We identified the date of each patient’s first visit to a medical institution (either outpatient or inpatient care) due to migraines as the major or minor diagnosis (ICD-10 code G43.x) during 2004–2015. That patient was defined as the migraine diagnosis group.

The major interesting variable was the diagnosis of sleep disorder. The Korean medical system must submit the patient’s medical records in order to receive compensation, and the patient's physician submit the patient’s data, including the patient’s diagnosis. Thus, all claim data includes major diagnoses diagnosed by each patient’s physician based on the ICD-code. In this study, the sleep disorder was defined as an organic sleep disorder (ICD-10: G47.x), including insomnia, hypersomnia, disorders of the sleep-wake schedule, sleep apnea, narcolepsy/cataplexy, and other sleep disorder. We excluded non-organic sleep disorders (F41.x).

This study considered the characteristics of patients suggested as factors related to migraine [34,35]. We included other independent variables as follows: sex, age, economic level, types of insurance coverage, Charlson comorbidity index (CCI), and region. The ages were originally classified into 5-year intervals, but later into 10-year intervals: less than 29 years, 30–39 years, 40–49 years, 50–59 years, 60–69 years, 70–79 years, and more than 80 years. CCI is a scored based on the patient’s complications, and increasing the score means that the patient’s severity is worse; it can predict of severity of each patient [36].

The economic level was defined based on the insurance premiums in the claim data. All South Koreans have National Health Insurance (NHI), and they pay an insurance premium based on their economic level, such as salary or property (generally about 7% of the salary was paid as a premium in 2017; this proportion changed every year). Thus, insurance premiums could estimate the economic level indirectly; it was classified as less than 30% (low income), 31–60%, 61–90%, and more than 91% (high income). The types of insurance coverage were divided into three types in South Korea. If patients were workers, employers in all workplaces, public officials, private school employees, continuously insured persons, or daily paid workers at construction sites, they were defined as NHI employees. Those with NHI employee insurance paid a regular portion of their average salary in contribution payments, and included spouses, dependents, siblings, and parents. The NHI self-employed insurance category included people who did not fall into the NHI employee insurance group. Their contribution amount was set by taking into account their income, property, living standard, and rate of participation in economic activities. Medical aid beneficiaries were defined as patients with an income below the government-defined poverty level, or those with a disability who were provided with free inpatient and outpatient care paid with government funds. Therefore, the type of insurance coverage represented each patient’s socioeconomic status. The CCI was calculated by weighing and scoring for comorbid conditions, with additional points added for comorbidities that could affect health outcomes of patients [37]. CCI was categorized into three groups as follows: ‘0’, ‘1’, ‘2’, ‘3’, and ‘more than 4’.

### 2.3. Ethical Consideration

This study was approved by the Institutional Review Board and the National Health Insurance Service Ilsan Hospital (NHIMC 2018-02-001).

### 2.4. Statistical Analysis

To investigate the association between migraines and sleep disorders, we first examined the frequencies and percentages of the study population according to the diagnosis of migraines. Next, we carried out a log-rank test and showed the Kaplan–Meier survival curve using a product-limit method to compare the rates of diagnosis for migraines between patients with or without sleep disorders. Third, we carried out a survival analysis using a Cox proportional hazard model with time-dependent covariates to identify the association between migraines and sleep disorders. In addition, to compare the risk of migraines according to the types of sleep disorder, using a detailed classification of sleep disorders such as insomnia, sleep apnea, and others, we carried out an additional survival analysis on the association with the incidence of migraines. Finally, to examine the differences in the association between migraines and sleep disorders, we also carried out sub-group analysis for survival analysis according to sex, age, economic level, and CCI. All statistical analyses were carried out using SAS statistical software version 9.2 (IBM, Cary, NC, USA).

## 3. Results

**Figure 1:**
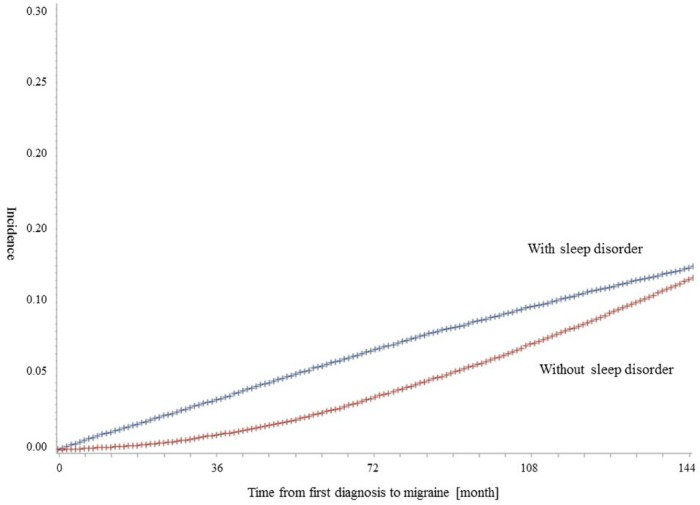
Kaplan–Meier survival curve for the incidence of migraine. The results of the log-rank test for time to migraine by the diagnosis of sleep disorder were statistically significant.

There were 133,262 patients (one with sleep disorder vs one without sleep disorder) during 2004–2015 in this study. Table 1 shows the distribution of the study population by the diagnosis of migraines. About 11.72% of patients were diagnosed with migraines. According to the diagnosis of sleep disorder, patients with sleep disorders had a diagnosis of migraine more frequently than patients without (yes. 12.04%; no, 11.39%; *p* = 0.0002). In addition, females, the elderly, those at a low economic level, with medical aid, or patients who lived in rural areas, were more frequently diagnosed as having migraines, and these associations were statistically significant. Figure 1 shows the results of the Kaplan–Meier survival curve and log-rank test to compare the rates of migraine according to the diagnosis of sleep disorder. The time-to-diagnosis of migraines was shorter for patients with sleep disorders than for those without.

Table 2 shows the results of the survival analysis to investigate the association between migraines and sleep disorders. Sleep disorder was positively correlated with diagnosis of migraine (yes: Hazard Ratio (HR), 1.591; 95% CI, 1.543–1.641; *p* < 0.0001; ref = no). In the results of other independent variables, female patients were more closely associated with a higher risk of migraines (female: HR, 1.833; 95% CI, 1.780–1.887; *p* < 0.0001; ref = male). In addition, the risk of diagnosis of migraine was greatest in the 40–49 years group. For the types of insurance coverage, the medical-aid group had a greater risk of migraines. By the severity of the comorbid condition, the patients with more comorbidity were more closely associated with migraines. Finally, patients who lived in rural areas were more positively associated with migraines (metropolitan: HR, 1.055; 95% CI, 1.020–1.092; *p* = 0.0017. Others: HR, 1.237; 95% CI, 1.200–1.275; *p* < 0.0001; ref = male).

Table 3 shows the results of additional survival analysis using detailed classifications of sleep disorder. By the types of sleep disorder, patients who were diagnosed with insomnia were more closely associated with migraines than with other types of sleep disorder or sleep apnea (insomnia: HR, 1.792; 95% CI, 1.740–1.846; *p* < 0.0001. Sleep apnea: HR, 1.250; 95% CI, 1.138–1.373; *p* < 0.0001. Other sleep disorders: HR, 1.663; 95% CI, 1.602–1.727; *p* < 0.0001).

**Figure 2:**
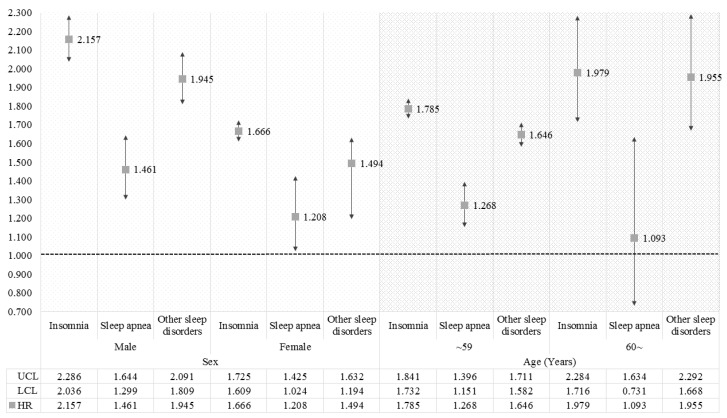
The results of sub-group analysis for survival analysis according to sex and age. The HR was calculated by survival analysis to investigate the association between sleep disorder and migraine. Results were considered to be statistically significant if each bar marked to SD did not reach the cut-off line of 1.000.

**Figure 3:**
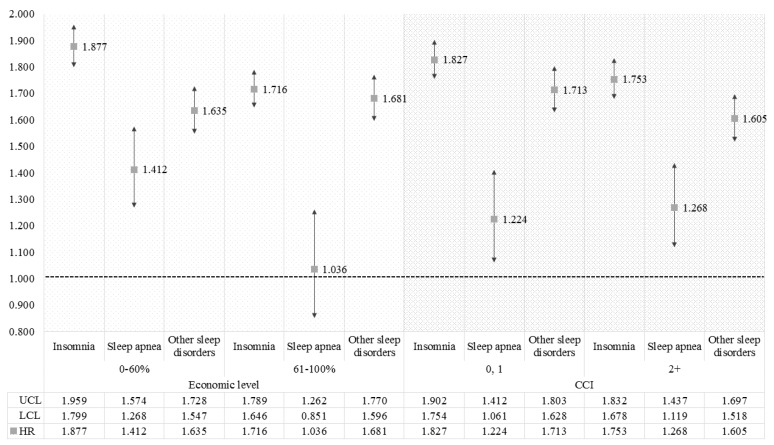
The results of the sub-group analysis for survival analysis according to the economic level and Charlson comorbidity index (CCI). The HR was calculated by survival analysis to investigate the association between sleep disorder and migraine. Results were considered statistically significant if each bar marked to SD did not reach the cut-off line of 1.000.

Figure 2 and Figure 3 show the results of sub-group analysis for survival analysis to identify the differences in association between types of sleep disorder and migraines according to sex, age, economic level, and CCI. For sex, the increasing risks of migraines for each type of sleep disorder were greater in male patients than in females. For the sub-group analysis on age, the higher age groups had greater effects from sleep disorders on the incidence of migraines, which are also generally greater in patients at a lower economic level than others. On the other hand, the results for the CCI showed a greater risk of migraines in patients without a comorbid condition, or with the lowest comorbid score.

## 4. Discussion

The aim of this study was to evaluate the association between sleep disorder and migraine. Our findings suggest that sleep disorders which ware caused by primary psychiatric problems could affect the incidence of migraines. Such a health outcome due to sleep disturbance is consistent with previous findings [28,33,38]. Our findings point toward a similar conclusion about sleep health, particularly in relation to migraines. Based on our findings, health care professionals and policy makers will need to find appropriate controls to take into account the health effects of sleep disorders.

In our study, the trends in the diagnosis of migraine in both sleep and general people increased over time during the study period. A possible explanation would be associated with the change in healthcare perception over a 10-year period. The development of the economic level has increased the awareness of people's health, and the increased interest in their health may lead to increased medical access, which may have led to an increase in the diagnosis of migraine in the general population [39].

Our findings were also interesting in terms of the detailed classification of sleep disorders and the sub-group analysis. Unlike previous findings, we distinguished the type of sleep disorder in analyzing for the association with migraines, and found that insomnia is a greater risk factor for migraines than are other types of sleep disorder. Thus, the lack of sleep duration could result in the loss of physical condition, not just the quality of sleep, and optimal alternatives are needed to ensure substantial sleep duration [40,41]. However, most people do not think that sleeping problems are more serious than other diseases such as cancer, and so they may not visit the hospital, even if they recognize that they have problems with sleeping [42]. In addition, if patients went to a medical institution because of a sleep disorder, they would face larger medical expenditures than for other diseases, because most sleep disorders were not covered under NHIS. As a result, the recent situation in sleep health will not be fully controlled at the individual level, and therefore, policy makers and insurers have to consider expanding the scope of insurance coverage to improve the economic accessibility of sleep medicine, considering other diseases, including migraines, that are associated with sleep disorders. Furthermore, people should be informed about the potential for sleep-related diseases, and patients with sleep disorders should be educated about appropriate management practices.

In addition, the sub-group analysis showed that associations with migraines were greater for males, the elderly population, and persons at a low economic level. It seemed that patients with less access to healthcare were more greatly exposed to the risk of migraines after sleep disorders. Thus, these results also confirmed the results about the low accessibility for managing sleep duration and quality; there is therefore a need to improve both insurance coverage and education for managing individual health. On the other hand, the results for the CCI showed that patients with mild comorbid conditions were highly associated with incidence of migraines after sleep disorders, perhaps because severe patients generally seek attention for their other severe symptoms, and not just for the sleep disorder. Patients with high severity will be more careful about their health, and manage their health to reduce other complications. However, patients with low comorbidity, that is, the general population, do not considered sleep problems seriously, because they think they will stay healthy. Differences in individual health care can lead to migraines in patients, and patients without proper management will have an increased burden of disease. Thus, early intervention and prevention will be required to prevent burdens due to sleep disorders.

Our study has some strengths. First, the data used in this study was a retrospective cohort study, and included the claim data of South Koreans under NHI. Thus, the results based on this data could be helpful in establishing health policies and creating alternatives based on the external validity, from the nature of the data. Second, there are many studies about sleep and health outcomes so far. However, most of them have been focused on severe outcomes, such as cerebrovascular disease or cancer. Others only contain findings related to psychiatric problems. To our best knowledge, this is the first attempt to investigate the association between sleep disorders and migraines using nationwide claim data. Although the severity of outcome variables is less than in previous studies, this study provides evidence of problems with sleep disorders that are also related to quality of life [43]. It will contribute to improving the quality of life by solving sleep problems. Third, this study used the matched cohort design using propensity scores in this study, to reduce selection bias and recall bias.

Despite these strengths, this study does have some limitations. Previous findings used the actual measuring data, such as duration of sleep or sleep tests. But our data only consisted of claim data, made for claiming medical costs. Thus, this study could not consider such information. Similarly, the medicine for curing sleep disorders would be a key factor in managing health, but most therapies for sleep disorder are not covered by NHIS, so we could not include them. Also, because it is a coding-based diagnosis, it is possible that potential diagnostic errors may have occurred, depending on the physician. In addition, various detailed sleep disorders can be classified as one of the other sleep disorders, potentially causing errors in our results. The relationship between various sleep disorders, classified as other sleep disorders, and migraine, may differ from our main findings. Finally, the severity of each disease was different in causing the outcome variable, but, in the process of using claim data, there were not enough variables for considering this. Thus, we used CCI to adjust each patient’s clinical condition.

Despite the several limitations related to the nature of claim data, our findings suggest that sleep disorders without a psychiatric disorder can be associated with migraines, in particular, insomnia. Its associations are greater for patients classified as male, low income, or elderly. On the other hand, such associations are more positively associated with migraines among patients with mild comorbid conditions. Based on these findings, healthcare professionals and policy makers need to review the present level of insurance coverage in sleep medicine, and educate patients for preventing unnecessary burdens due to sleep disorders.

## 5. Conclusion

Migraines can be associated with primary sleep disorders, in particular, insomnia. Its associations were greater for males, persons with lower income, the elderly population, and patients with mild comorbid conditions. Thus, there is a need to review the present strategies related to sleep disorders, and to provide optimal information about sleep health to the general population. 

## Figures and Tables

**Table 1:** General characteristics of the study population.

Variables	Total		Migraine				*p*-Value
			Diagnosed		None		
	*N*	%	*N*	%	*N*	%	
Sleep disorder							
Yes	66,631	50.00	8025	12.04	58,606	87.96	0.0002
No	66,631	50.00	7591	11.39	59,040	88.61	
Sex							
Male	55,614	41.73	4293	7.72	51,321	92.28	<0.0001
Female	77,648	58.27	11,323	14.58	66,325	85.42	
Age (Years)							
~29	6199	4.65	471	7.60	5728	92.40	<0.0001
30–39	12,124	9.10	1290	10.64	10,834	89.36	
40–49	18,563	13.93	2148	11.57	16,415	88.43	
50–59	26,726	20.06	3374	12.62	23,352	87.38	
60–69	25,794	19.36	3059	11.86	22,735	88.14	
70–79	23,717	17.80	3182	13.42	20,535	86.58	
80~	20,139	15.11	2092	10.39	18,047	89.61	
Economic level							
~30% (low)	36,151	27.13	4408	12.19	31,743	87.81	0.0001
31–60%	33,177	24.90	3919	11.81	29,258	88.19	
61–90%	44,898	33.69	5215	11.62	39,683	88.38	
+91% (high)	19,036	14.28	2074	10.90	16,962	89.10	
Types of insurance coverage							
Medical aid	6733	5.05	932	13.84	5801	86.16	<0.0001
NHI, self-employed insured	54,054	40.56	6492	12.01	47,562	87.99	
NHI, employee insured	72,475	54.39	8192	11.30	64,283	88.70	
Charlson comorbidity index							
0	109,022	81.81	12,596	11.55	96,426	88.45	<0.0001
1	15,257	11.45	1994	13.07	13,263	86.93	
2	6015	4.51	693	11.52	5322	88.48	
3	1165	0.87	130	11.16	1035	88.84	
4+	1803	1.35	203	11.26	1600	88.74	
Region							
Capital area	55,652	41.76	5971	10.73	49,681	89.27	<0.0001
Metropolitan	33,888	25.43	3848	11.36	30,040	88.64	
Others	43,722	32.81	5797	13.26	37,925	86.74	
Total	133,262	58.24	15,616	11.72	117,646	88.28	

**Table 2:** Results of survival analysis for the association between sleep disorder and migraine.

Variables	Migraine			
	HR	95% CI		*p*-Value
		Lower	Upper	
Sleep disorder				
Yes	1.591	1.543	1.641	<0.0001
No	1.000	—	—	—
Sex				
Male	1.000	—	—	—
Female	1.833	1.780	1.887	<0.0001
Age (Years)				
~29	1.000	—	—	—
30–39	1.450	1.335	1.575	<0.0001
40–49	4.578	1.460	1.707	<0.0001
50–59	1.690	1.567	1.822	<0.0001
60–69	1.682	1.559	1.816	<0.0001
70–79	1.898	1.759	2.048	<0.0001
80~	1.625	1.500	1.761	<0.0001
Economic level				
~30% (low)	1.000	—	—	—
31–60%	1.035	0.988	1.083	0.1447
61–90%	1.069	1.022	1.117	0.0035
91%~ (high)	1.020	0.977	1.064	0.3727
Types of insurance coverage				
Medical aid	1.251	1.175	1.330	<0.0001
NHI, self-employed insured	1.011	0.984	1.039	0.4098
NHI, employee insured	1.000	—	—	—
Charlson comorbidity index				
0	1.000	—	—	—
1	1.151	1.107	1.197	<0.0001
2	1.111	1.047	1.179	0.0005
3	1.090	0.954	1.245	0.2036
4+	1.164	1.049	1.291	0.0041
Region				
Capital area	1.000	—	—	—
Metropolitan	1.055	1.020	1.092	0.0017
Others	1.237	1.200	1.275	<0.0001

**Table 3:** Results of survival analysis for association between types of sleep disorder and migraine.

Variables	Migraine			
	HR	95% CI		*p*-Value
		Lower	Upper	
Sleep disorder				
Insomnia	1.792	1.740	1.846	<0.0001
Sleep apnea	1.250	1.138	1.373	<0.0001
Other sleep disorders	1.663	1.602	1.727	<0.0001
None	1.000	—	—	—
Sex				
Male	1.000	—	—	—
Female	1.810	1.758	1.864	<0.0001
Age (Years)				
~29	1.000	—	—	—
30–39	1.470	1.353	1.597	<0.0001
40–49	1.603	1.483	1.733	<0.0001
50–59	1.716	1.592	1.851	<0.0001
60–69	1.723	1.596	1.859	<0.0001
70–79	1.944	1.802	2.098	<0.0001
80~	1.683	1.554	1.823	<0.0001
Economic level				
~30% (low)	1.000	—	—	—
31–60%	1.032	0.986	1.080	0.1734
61–90%	1.071	1.024	1.120	0.0025
91%~ (high)	1.025	0.982	1.070	0.2510
Types of insurance coverage				
Medical aid	1.228	1.154	1.306	<0.0001
NHI, self-employed insured	0.999	0.972	1.027	0.9436
NHI, employee insured	1.000	—	—	—
Charlson comorbidity index				
0	1.000	—	—	—
1	1.125	1.082	1.170	<0.0001
2	1.027	0.968	1.090	0.3745
3	1.024	0.896	1.170	0.7254
4+	1.042	0.939	1.156	0.4372
Region				
Capital area	1.000	—	—	—
Metropolitan	1.054	1.019	1.090	0.0021
Others	1.238	1.201	1.277	<0.0001
